# mTORC1-Activated Monocytes Increase Tregs and Inhibit the Immune Response to Bacterial Infections

**DOI:** 10.1155/2016/7369351

**Published:** 2016-09-26

**Authors:** Lijun Fang, Huaijun Tu, Wei Guo, Shixuan Wang, Ting Xue, Fei Yang, Xiaoyan Zhang, Yazhi Yang, Qian Wan, Zhexin Shi, Xulong Zhan, Jian Li

**Affiliations:** ^1^Department of Hematology, The Second Affiliated Hospital of Nanchang University, Key Laboratory of Experimental Hematology in Jiangxi Province, Nanchang, Jiangxi, China; ^2^Medical Department of Nanchang University Graduate School, Nanchang, Jiangxi, China; ^3^Department of Neurology, The Second Affiliated Hospital of Nanchang University, Nanchang, Jiangxi, China; ^4^State Key Laboratory of Experimental Hematology, Institute of Hematology and Hospital of Blood Disease, Chinese Academy of Medical Sciences, Tianjin, China; ^5^Department of Medical Microbiology, Tongji Medical College, Huazhong University of Science and Technology, Wuhan, Hubei, China; ^6^Department of Hematology and Oncology, The First Teaching Hospital of Tianjin University of Traditional Chinese Medicine, Tianjin, China

## Abstract

The TSC1/2 heterodimer, a key upstream regulator of the mTOR, can inhibit the activation of mTOR, which plays a critical role in immune responses after bacterial infections. Monocytes are an innate immune cell type that have been shown to be involved in bacteremia. However, how the mTOR pathway is involved in the regulation of monocytes is largely unknown. In our study, TSC1 KO mice and WT mice were infected with* E. coli*. When compared to WT mice, we found higher mortality, greater numbers of bacteria, decreased expression of coactivators in monocytes, increased numbers of Tregs, and decreased numbers of effector T cells in TSC1 KO mice. Monocytes obtained from TSC1 KO mice produced more ROS, IL-6, IL-10, and TGF-*β* and less IL-1, IFN-*γ*, and TNF-*α*. Taken together, our results suggest that the inhibited immune functioning in TSC1 KO mice is influenced by mTORC1 activation in monocytes. The reduced expression of coactivators resulted in inhibited effector T cell proliferation. mTORC1-activated monocytes are harmful during bacterial infections. Therefore, inhibiting mTORC1 signaling through rapamycin administration could rescue the harmful aspects of an overactive immune response, and this knowledge provides a new direction for clinical therapy.

## 1. Introduction

Tuberous sclerosis complex (TSC) is an autosomal dominant disorder that results from mutations in the TSC genes,* TSC1* and* TSC2*. The heterodimer, which consists of TSC1 and TSC2 and is encoded by* TSC1* and* TSC2*, respectively, is a key upstream regulator of the mammalian target of rapamycin (mTOR) pathway [1]. Mutations in the TSC1/2 genes could impair the inhibitory function of the TSC1/2 complex, leading to mTOR signaling activation and downstream effector phosphorylation, such as p70 S6 kinase (S6K) and 4E-binding protein 1 (4EBP1), which ultimately affect cellular processes. Recently, mTORC1 has been found to play a critical role in the immune response to bacterial infections [2, 3]. In response to lipopolysaccharide (LPS), a Toll-like receptor (TLR) stimulator, protein synthesis is rapidly enhanced in mouse macrophages and dendritic cells, which is largely mediated by the phosphatidylinositol 3-kinase– (PI3K–) mTORC1 pathway [4, 5]. In the murine macrophage-like cell line and a mouse model of bacterial infection, immune activation was also observed after treatment with rapamycin (Rapa), the mTORC1 pathway inhibitor [5, 6]. PI3K signaling could now be targeted to determine its contribution to rheumatoid and psoriatic arthritis where deregulated proliferation and aberrant survival of activated immune cells, macrophages, monocytes, dendritic cells, and synovial fibroblasts significantly overlap with abnormal growth of cancer cells [7].

Bacteremia, a common bacterial disease, involves multiple immune processes, such as immune cell activation and disordered cytokines expression [8]. When host immune mechanisms eliminate bacteria from the blood, it is usually transient and clinically benign. However, when host mechanisms fail, bacteremia, and even sepsis, can result [9]. Monocytes, a type of innate immune cell [10], have been shown to be involved in bacteremia [11]. Although the regulatory mechanisms of monocyte activity are not fully understood, the monocytic innate immune response has been reported in several studies [11]. Inflammatory monocytes were observed to egress from the bone marrow to the bloodstream where they released trypanotoxic molecules (i.e., TNF and NO) [12]. Monocytes have also been shown to participate in T cell priming and polarization [13, 14]. Inflammatory monocytes also influence Th1 and Th17 differentiation by inducing T-bet expression [14]. In THP-1-derived macrophages and human monocytes, mTOR inhibition by rapamycin reversed* L. donovani*-induced IL-12 and IL-10 modulation.* L. donovani* induced phosphorylation of P70S6K, a correlate of mTOR activity, in TLR-stimulated THP-1 derived macrophages [15]. In human dendritic cells, mTOR regulates IL-12 p40-mediatied Th1(IFN-*γ*) and Th17(IL-17) responses in autoimmune disease [16]. However, how the mTORC1 pathway regulates monocytes in bacterial infections is largely unknown. In this study, we aim to investigate how the mTORC1 pathway regulates the functions of peripheral blood monocytes in the early phase of bacteremia.

## 2. Materials and Methods

### 2.1. Animals and Treatments

*Tsc1* ^flox/flox^ mice and mice expressing Cre recombinase (a tyrosine recombinase enzyme) were purchased from Jackson Laboratories (Bar Harbor, USA). Mice with a homozygous* Tsc1* deletion in myeloid cells (LysMCre*Tsc1* ^flox/flox^; TSC1 KO) were obtained by crossing two mice expressing Cre under the control of lysozyme promoter (LysMCre). LysMCre-negative,* Tsc1* ^flox/flox^ mice served as controls (WT mice). Rosa26R-EYFP mice were purchased from Jackson Laboratories (Bar Harbor, USA) and bred to LysMCre*Tsc1* ^flox/flox^ mice to monitor Cre expression using enhanced yellow fluorescent protein (EYFP) expression. EYFP was observed in Cre-expressing tissues of the double mutant offspring (Rosa-LysMCre*Tsc1* ^flox/flox^). All mice were maintained in a specific pathogen-free facility and were used in accordance with protocols approved by Animals Care and User Committee at the Institute of Hematology, Peking Union Medical College.

Rapamycin (Rapa) (Cayman Chemical, Country) was dissolved in PBS containing 5% DMSO. Mice aged 6–8 weeks were intraperitoneally injected with Rapa (1.5 mg/kg) every day for four days.

### 2.2. Flow Cytometry

Lymph node and spleen cells were isolated from KO and WT mice. Briefly, 6- to 8-week-old male and female mice were sacrificed, and peripheral lymph nodes (pLNs), mesenteric LNs (mLNs), and spleens were resected and transferred to PBS containing 2% FBS. Lymph nodes (LNs) and spleen cells were obtained by filtering LNs and spleen cells through a 48-micron nylon mesh. Approximately one million white blood cells were incubated with antibodies (anti-CD4-FITC) in the dark for 30 min and then washed with staining buffer. After staining with anti-CD4-FITC, anti-Ki67-APC, anti-IL-17-PE, anti-Foxp3-PE/Cy7, and anti-IFN-*γ*-PerCP/Cy5.5, intracellular staining was performed using a Mouse Regulatory T Cell Staining Kit (eBioscience).

### 2.3. *E. coli* Infection

Fifty million* E. coli* cells (ATCC, USA) were injected through the tail vein for infection of mice. Eight hours postinfection, peripheral blood was collected by retroorbital puncture and placed into EDTA-containing collection tubes. Blood samples were treated with red blood cells lysis buffer and washed once with PBS containing 2% FBS. Cell lysate was then centrifuged at 3000 rpm for 10 min.

*Assessment of Residual Bacteria Amount*. Cell supernatants were diluted 20-fold with LB medium and grown at 37°C at 200 rpm for 14 hours. Growth was monitored by absorbance (OD600), where OD = 1 corresponded to 10^9^ cells/mL. Approximately one million white blood cells were incubated with antibodies (anti-CD11b-PE/Cy7, anti-Ly6G-APC/Cy7, anti-Ly6C-PerCP/Cy5.5, anti-CD80-PE/Cy7, CD40-PE/Cy7, anti-CD86-PE, anti-CD14-FITC, anti-MHC-II-APC, and anti-TLR4-PE) in the dark for 30 min and then washed with staining buffer. Cells were measured using a flow cytometer (BD FACSCanto II, USA). The data were analyzed using FlowJo software (Treestar, USA).

*Assessment of Various Cytokines Plasma Concentrations*. The rest of the cell supernatants was stored at −80°C. Concentrations of TNF*α*, IL-1*β*, IL-6, IL-10, IFN-*γ*, and TGF-*β* were measured by QuantiCyto® ELISA kit (NeoBioscience, Shenzhen, China) according to the manufacturer's instructions.

*Flow Cytometry*. Male and female mice were sacrificed, and pLNs, mLNs, and spleens were resected and transferred to PBS containing 2% FBS. The LNs and spleens cells were obtained by filtering LNs and spleens through a 48-micron nylon mesh. Approximately one million cells were incubated with antibodies (anti-CD4-APC, anti-CD62L-FITC, and anti-CD44-PerCP/Cy5.5) in the dark for 30 min and then washed with staining buffer. Cells were measured using a flow cytometer (BD FACSCanto II, USA). The data were analyzed using FlowJo software (Treestar, USA).

### 2.4. Cellular Coculture Experiments

*Proliferation Experiment*. Lymph node cells were isolated from WT mice at 6–8 weeks of age. After sacrifice, pLNs and mLNs were resected and transferred to PBS containing 2% FBS. Lymph node cells were obtained by filtering pLNs and mLNs through a 48-micron nylon mesh. Regulatory T cells (Tregs, CD4^+^CD25^+^) and naïve CD4^+^ T cells (CD4^+^CD44^−^CD62L^+^) from lymph nodes were sorted and incubated at a concentration of 10^7^ cells/mL with 2 *μ*M carboxyfluorescein succinimidyl ester (Invitrogen, USA) for 10 min at room temperature and washed twice with PBS containing 5% FBS. Then, 2 × 10^5^ Tregs or naïve CD4^+^ T cells were cocultured with 1 × 10^5^ sorted monocytes (CD11b^+^Ly6G^−^Ly6C^+^) from WT or TSC1 KO mice in DMEM containing IL-2 (100 ng/mL) or anti-mouse CD3e (10 *μ*g/mL, BD Biosciences, USA) and CD28 antibody (10 *μ*g/mL, BD Biosciences, USA) in 96-well flat-bottomed plates. After 6 days, cells treated with IL-2 or CD3e and CD28 were used to estimate Tregs, CD4^+^ T, and Th17 cells by flow cytometry (BD FACSCanto II, USA).

*IL-10 Treatment Experiment*. Two hundred thousand sorted naïve CD4^+^ T cells (CD4^+^CD44^−^CD62L^+^) were cocultured with 1 × 10^5^ sorted monocytes (CD11b^+^Ly6G^−^Ly6C^+^) from WT mice in DMEM containing anti-mouse CD3e and CD28 antibody in 96-well flat-bottomed plates. Cells were treated with IL-10 (20 ng/mL, Peprotech, UK) or PBS. After 6 days, IL-17A secretion from CD4^+^ T cells was assayed by flow cytometry (BD FACSCanto II, USA).

### 2.5. Reactive Oxygen Species (ROS) Measurement

Peripheral blood samples from WT or TSC1 KO mice were treated with red blood cell lysis buffer. Approximately one million white blood cells were incubated with antibodies (anti-mCD11b-PE/Cy7, anti-mLy6C-PerCP-Cy5.5, and anti-mLy6G-APC/Cy7) in the dark for 30 min and then washed with staining buffer. After incubation with 10 *μ*M CM-H2DCFDA (Invitrogen, USA) at 37°C for 30 min, ROS was measured using flow cytometry (BD FACSCanto II, USA). The data were analyzed using FlowJo software (Treestar, USA).

### 2.6. RT-PCR

CD11b^+^Ly6G^−^Ly6c^+^ cells were sorted by BD FACSAria III. Total RNA was isolated using TRIzol reagent (Invitrogen, USA). Reverse transcription was performed with M-MLV reverse transcriptase (TransGen Biotech) according to the manufacturer's instructions. RT-PCR was determined using SYBR Green Master Mix (Life Technologies™). The cycling threshold value for GAPDH was subtracted from the cycling threshold (ΔCt). Relative gene quantification was performed using the comparative 2^−ΔΔCt^ method and normalized to GAPDH. The primers used for PCR were as follows: forward 5′-CTTTGTCAAGCTCATTTCCTGG-3′ and reverse 5′-TCTTGCTCAGTGTCCTTGC-3′ (GAPDH); forward 5′-CAAAGCCAGAGTCCTTCAGAG-3′ and reverse 5′-GTCCTTAGCCACTCCTTCTG-3′ (IFN-*γ*); forward 5′-ACGGACCCCAAAAGATGAAG-3′ and reverse 5′-TTCTCCACAGCCACAATGAG-3′ (IL-1*β*); forward 5′-GAGTGACAAGCCTGTAGCC-3′ and reverse 5′-CTCCTGGTATGAGATAGCAAA-3′ (TNF-*α*); forward 5′-GAGACGGAATACAGGGCTTTC-3′ and reverse 5′-TCTCTGTGGAGCTGAAGCAAT-3′ (TGF-*β*); forward 5′-AGCCGGGAAGACAATAACTG-3′ and reverse 5′-GGAGTCGGTTAGCAGTATGTTG-3′ (IL-10); forward 5′-CAAAGCCAGAGTCCTTCAGAG-3′ and reverse 5′-GTCCTTAGCCACTCCTTCTG-3′ (IL-6).

### 2.7. Statistical Analysis

Data are presented as the mean ± standard error of the mean (SEM). Unpaired Student's *t*-test was used to evaluate the difference between the two groups. A two-tailed *P* value of less than 0.05 was considered statistically significant (^*∗*^ *P* < 0.05, ^*∗∗*^ *P* < 0.01, and ^*∗∗∗*^ *P* < 0.001).

## 3. Results

### 3.1. *E. coli* Infection Activated the mTORC1/S6k Pathway in Monocytes, and Infection of TSC1 KO Mice Partly Simulated* E. coli* Infection

**Figure 1:**
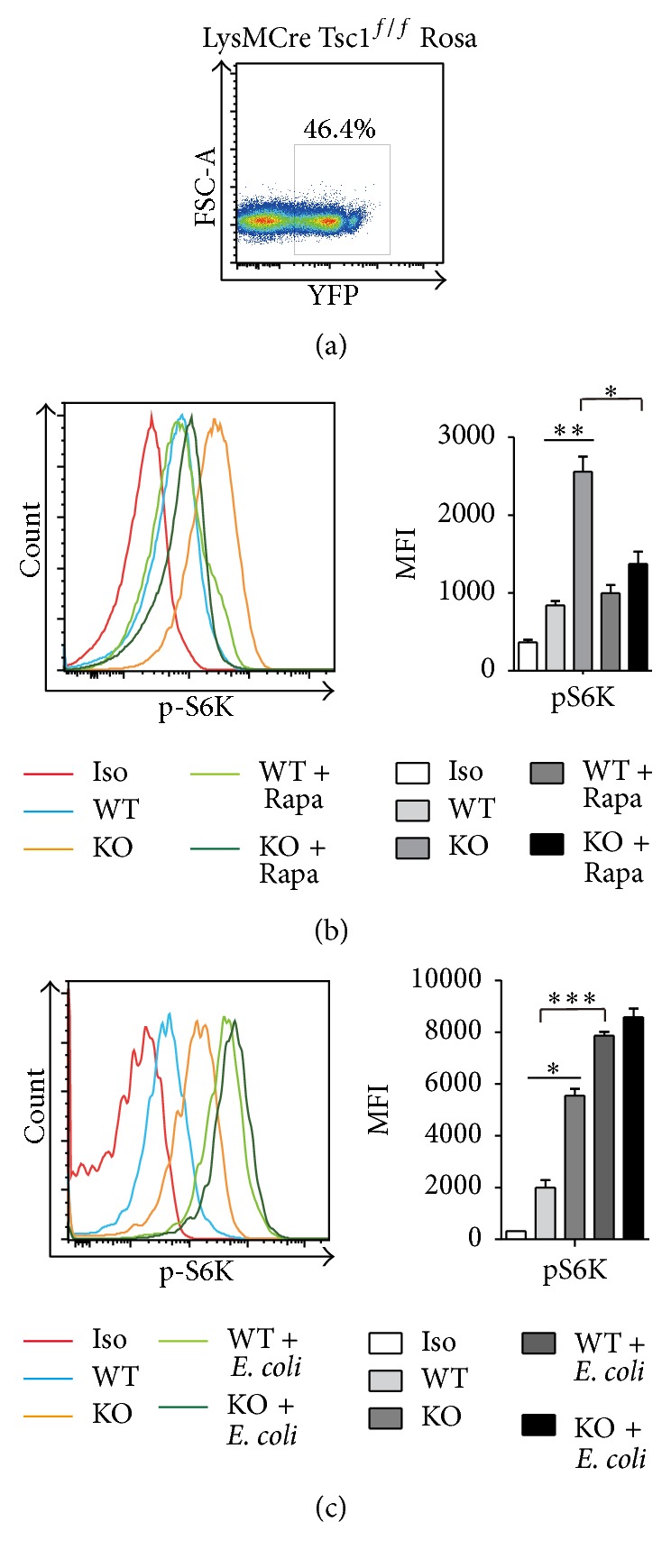
*E. coli* infection activated mTORC1/S6k pathway in monocytes, and TSC1 KO mice partly simulated* E. coli* infection. (a) The percentage of EYFP in monocytes from Rosa-LysMCreTsc1^flox/flox^ mice. This percentage represents the TSC1 deletion ratio in TSC1 KO monocytes. (b) The phosphorylation level of S6k in WT and TSC1 KO monocytes (CD11b^+^Ly6C^+^Ly6G^−^) in the presence or absence of Rapa (1.5 mg/kg, four days before treatment) (MFI: median fluorescence intensity) (three mice per group, three representative experiments). (c) The phosphorylation level of S6k in WT and TSC1 KO monocytes (CD11b^+^Ly6C^+^Ly6G^−^) with or without* E. coli* infection (5*∗*10^7^ cells/mouse) for 8 h (three mice per group, three representative experiments). ^*∗*^ *P* < 0.05, ^*∗∗*^ *P* < 0.01, and ^*∗∗∗*^ *P* < 0.001 compared to WT mice or between the indicated groups. *P* values were determined using Student's *t*-tests.

We used LysMCre* Tsc1* ^*f/f*^Rosa mice to detect the ratio of* Tsc1* knockout monocytes. The percentage of YFP represents the ratio of Tsc1 knockout monocytes (Figure 1(a)). S6K is the downstream effector of mTORC1 pathway, and its phosphorylation (pS6K) represents activation of mTORC1 pathway. The expression of pS6K in monocytes obtained from WT, TSC1 KO, and TSC1 KO mice treated with Rapa (KO + Rapa) indicated that the absence of* Tsc1* can activate mTORC1, and activation of the mTORC1 pathway can be rescued by Rapa (Figure 1(b)). When mice were infected with* E. coli*, the expression of pS6K was also increased in monocytes of WT mice (WT +* E. coli*), indicating that* E. coli* infection activates mTORC1 pathway in monocytes and mTORC1-activated monocytes in bacterial infection can be simulated partly by TSC1 KO mice (Figure 1(c)).

### 3.2. TSC1 KO Mice Displayed Greater Numbers of Bacteria in the Blood and Greater Mortality with* E. coli* Infection

**Figure 2:**
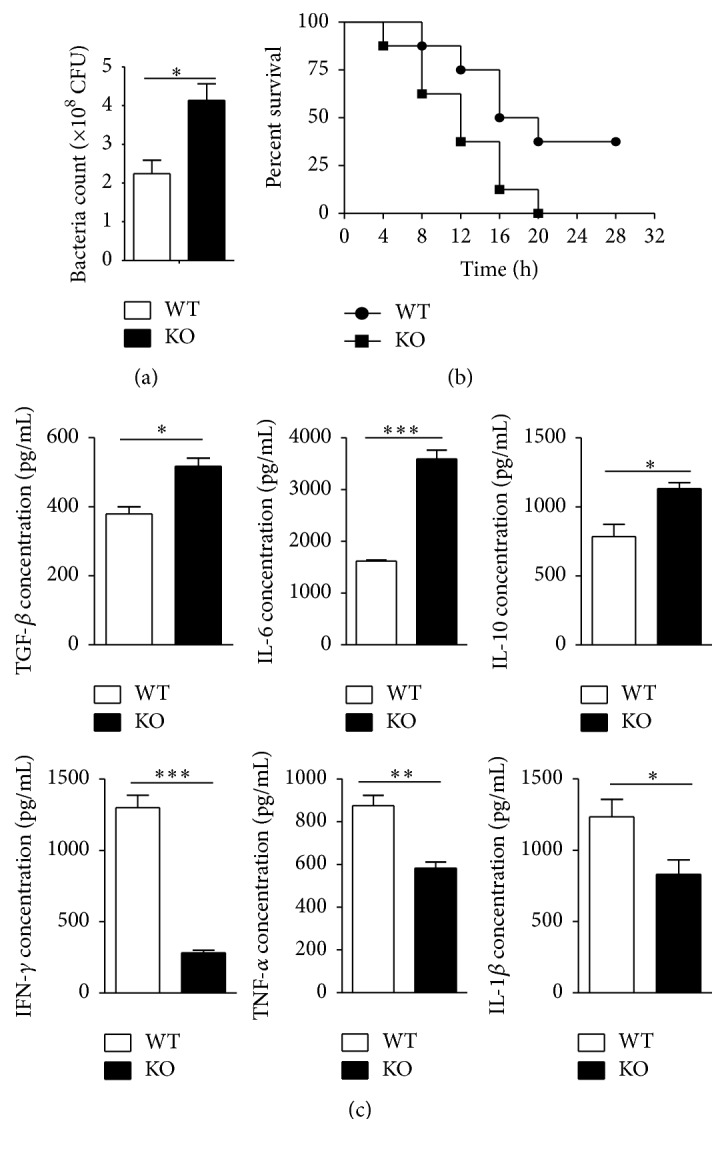
Bacterial number in the blood and survival curves of mice infected with* E. coli*. (a) The residual bacteria number in TSC1 KO mice is higher than in WT mice after* E. coli* infection (5*∗*10^7^ cells/mouse) (three mice per group, two representative experiments). (b) The survival curves of mice after* E. coli* infection (6–8 mice per group, four representative experiments). (c) The concentration of various cytokines in the serum of WT and TSC1 KO mice after 6 h bacterial infection by ELISA. ^*∗*^ *P* < 0.05, ^*∗∗*^ *P* < 0.01, and ^*∗∗∗*^ *P* < 0.001 compared to WT mice or between the indicated groups. *P* values were determined using Student's *t*-tests.

To compare resistance to bacterial infection in WT and TSC1 KO mice, we infected WT and TSC1 KO mice with* E. coli* (5*∗*10^5^ cells/mouse). At eight hours postinfection, we collected peripheral blood and measured the amount of residual bacteria. We found significantly greater numbers of bacteria in TSC1 KO mice than in WT mice (Figure 2(a)). In addition, we recorded the survival time of mice after bacterial infection and plotted survival curves. The results showed a higher mortality in TSC1 KO mice compared to WT mice (Figure 2(b)). We also measured the levels of various cytokines in the serum of WT and TSC1 KO mice infected with bacteria by ELISA (Figure 2(c)) and found that IL-6, IL-10, and TGF-*β* increased and IFN-*γ*, IL-1*β*, and TNF-*α* decreased in TSC1 KO mice. These results suggested that, compared to WT mice, TSC1 KO mice exhibit a weakened immune response after bacterial infection.

### 3.3. The Expression of IL-6, TGF-*β*, and IL-10 Is Increased, While the Expression of IFN-*γ*, IL-1*β*, and TNF-*α* Is Decreased in Monocytes of TSC1 KO Mice with Infected Bacteria

**Figure 3:**
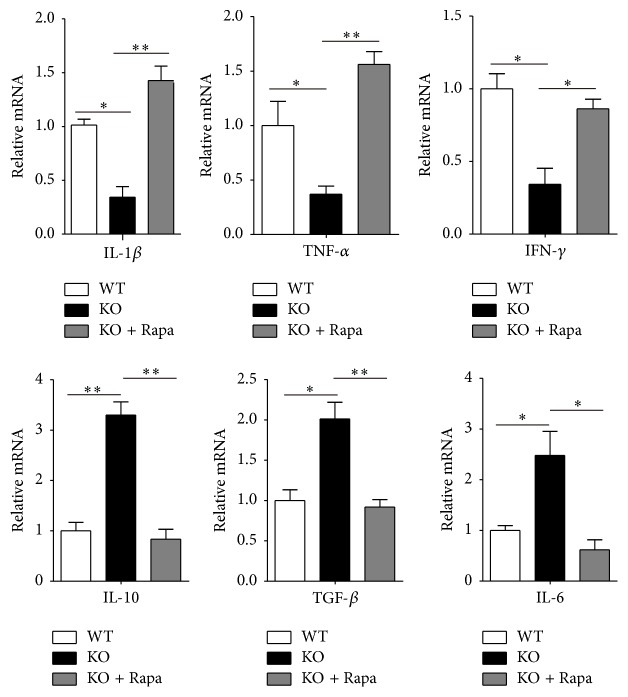
Change in cytokine expression by monocytes in mice with infected bacteria. Freshly isolated peritoneal monocytes (CD11b^+^Ly6G^−^Ly6C^+^) were sorted from WT, TSC1 KO, and TSC1 + Rapa (1.5 mg/kg, four days before treatment) mice after* E. coli* infection (5*∗*10^7^ cells/mouse), and the expression of TNF-*α*, IFN-*γ*, IL-1*β*, IL-10, and TGF-*β* was determined by real-time PCR (*n* = 3 representative experiments). ^*∗*^ *P* < 0.05 and ^*∗∗*^ *P* < 0.01 compared to WT mice or between the indicated groups. *P* values were determined using Student's *t*-tests.

To understand the TSC1 KO monocyte cytokine response, we evaluated the expression of cytokines from monocytes in mice with bacteria and found that monocytes (CD11b^+^Ly6C^+^Ly6G^−^) obtained from TSC1 KO mice had increased IL-6, IL-10, and TGF-*β*. This is in contrast to inflammatory cytokines, including IFN-*γ*, IL-1*β*, and TNF-*α*, that showed decreased expression when compared to WT mice (Figure 3). With Rapa treatment, IL-6, IL-10, and TGF-*β* decreased, while IFN-*γ*, IL-1*β*, and TNF-*α* increased in TSC1 KO mice. These results reveal that cytokine expression is regulated by mTORC1 signaling in monocytes in bacterial infection.

### 3.4. Tregs Increased While Th17 Decreased in the Lymph Nodes and Spleens of TSC1 KO Mice

**Figure 4:**
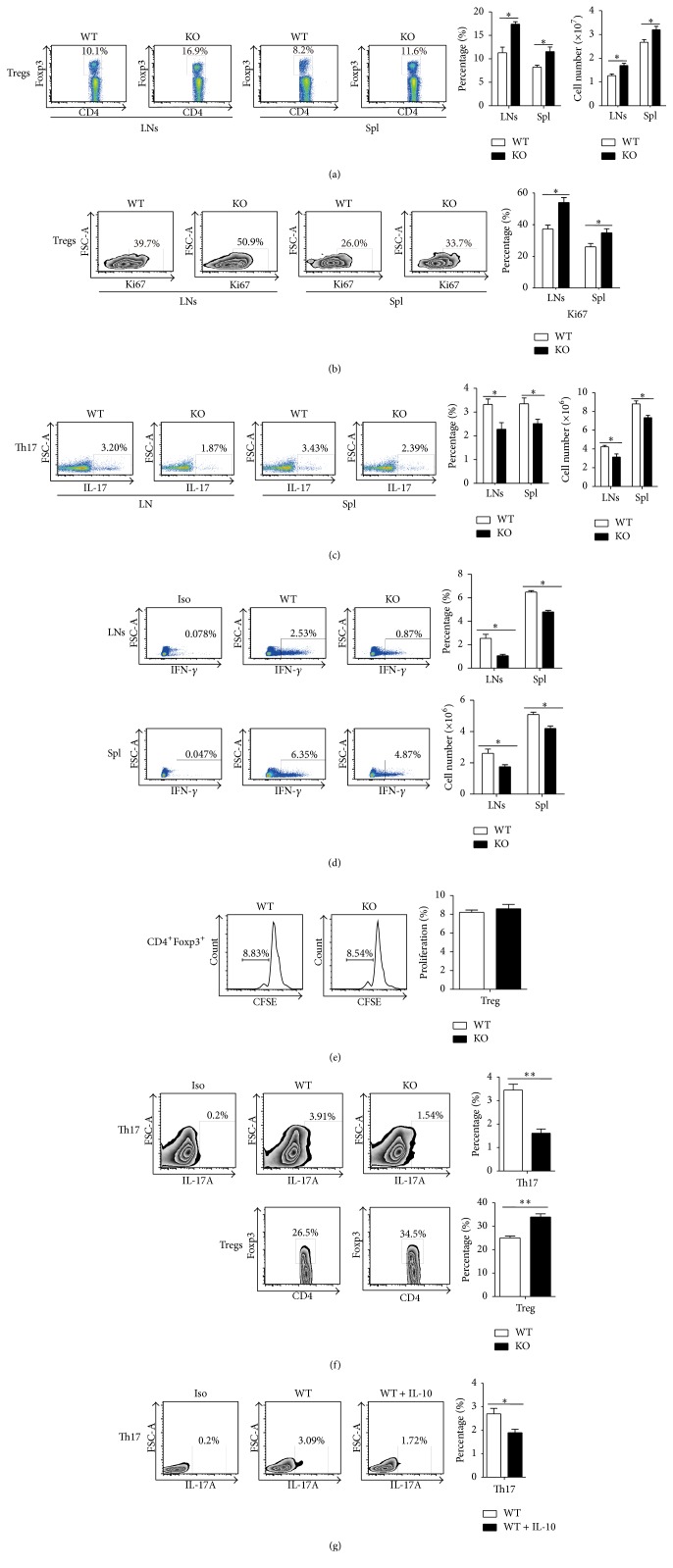
mTORC1 influences the numbers of Tregs and Th17 and Th1 cells in lymph nodes and spleen. (a) The percentage and number of Tregs increased in TSC1 KO lymph nodes and spleens compared to WT mice (four mice per group, three representative experiments). (b) The percentage of Ki67-positive Tregs increased in TSC1 KO lymph nodes and spleens compared to WT mice (four mice per group, three representative experiments). (c) The percentage and number of Th17 cells (CD4^+^IL-17^+^) decreased in lymph nodes and spleens in TSC1 KO mice when compared to WT mice (four mice per group, three representative experiments). (d) The number and percentage of Th1 cells (CD4^+^IFN-gamma^+^) decreased in the LNs and spleen of TSC1 KO mice compared to those of WT mice (four mice per group, two representative experiments). (e) 2 × 10^5^ Tregs (CD4^+^CD25^+^) labeled with CFSE (5 *μ*M; eBioscience) were cocultured with 1 × 10^5^ sorted monocytes (CD11b^+^Ly6G^−^Ly6C^+^) from WT or TSC1 KO mice in DMEM containing IL-2 (100 ng/mL) in 96-well flat-bottomed plates for 6 days. There was no significant difference in proliferation of Tregs cocultured with monocytes obtained from TSC1 KO or WT mice (*n* = 3 representative experiments). (f) 2 × 10^5^ naïve CD4^+^ T cells (CD4^+^CD44^−^CD62^+^) were cocultured with 1 × 10^5^ sorted monocytes (CD11b^+^Ly6G^−^Ly6C^+^) from WT or TSC1 KO mice in DMEM with anti-mouse CD3e (10 *μ*g/mL, BD Biosciences, USA) and CD28 antibody (10 *μ*g/mL, BD Biosciences, USA) in 96-well flat-bottomed plates for 6 days. The percentage of Th17 cells of naïve CD4^+^ T cells (CD4^+^CD44^−^CD62L^+^) cocultured with monocytes obtained from TSC1 KO mice was lower than the WT group at day 6. However, the percentage of Tregs was higher in the TSC1 KO group than in the WT group at day 6 (*n* = 3 representative experiments). (g) 2 × 10^5^ sorted naïve CD4^+^ T cells (CD4^+^CD44^−^CD62L^+^) were cocultured with 1 × 10^5^ sorted monocytes (CD11b^+^Ly6G^−^Ly6C^+^) from WT mice in DMEM with or without IL-10 (20 ng/mL, Peprotech, UK). After 6 days, cells were used to estimate Th17 cells using flow cytometry (BD FACSCanto II, USA). ^*∗*^ *P* < 0.05 and ^*∗∗*^ *P* < 0.01 compared to WT mice or between the indicated groups. *P* values were determined using Student's *t*-tests.

Because of the greater numbers of bacteria, lower concentration of proinflammatory cytokines, and greater mortality in TSC1 KO mice, we thought that TSC1 KO mice had an impaired antibacterial immune response, which leads to a weak immune response by TSC1 KO mice. Hence, we detected the numbers and proliferation of Tregs, whose function is to inhibit immune responses [17]. We found that the number and percentage of Tregs in the lymph nodes (LNs) and spleen (Figure 4(a)) are significantly higher in TSC1 KO mice than in WT mice, with a higher percentage of Ki67-positive Tregs (Figure 4(b)). Moreover, we also examined the number of Th17 cells, which balances the immune response against Tregs [18], and found that the number and percentage of Th17 cells decreased in the LNs and spleen of TSC1 KO mice than in those of WT mice (Figure 4(c)). Th1 are effective in providing protection against bacterial infections. We also detected the population of Th1 effector cell, finding that the number and percentage of Th1 cells decreased in the LNs and spleen of TSC1 KO mice compared to those of WT mice (Figure 4(d)). To determine if the increase of Tregs was induced directly by monocytes in TSC1 KO mice, we sorted monocytes (CD11b^+^Ly6C^+^Ly6G^−^) from WT and TSC1 KO mice and cocultured them with Tregs (CD4^+^CD25^+^) or CD4^+^ naïve T cells (CD4^+^CD62L^+^CD44^−^) obtained from WT mice. No significant difference in proliferation was observed in Tregs cocultured with monocytes obtained from TSC1 KO or WT mice (Figure 4(e)). However, when compared to WT mice, there was a lower percentage of Th17 cells and a higher percentage of Tregs in CD4^+^ naïve T cells that were cocultured with monocytes from TSC1 KO mice (Figure 4(f)). These findings indicated that the increase in Tregs was indirectly induced by monocytes, and it may be involved in the balance of Tregs to Th17 cells. When considering the changes in cytokine expression by TSC1 KO monocytes, we speculated that the decrease in Th17 cells may be related to the changes in cytokines. We treated WT monocytes cocultured with naïve CD4^+^ T cells with or without IL-10 treatment and found that IL-10 could decrease the percentage of Th17 cells (Figure 4(g)).

### 3.5. Greater Amounts of ROS Produced by Monocytes from TSC1 KO When Compared to WT Mice

**Figure 5:**
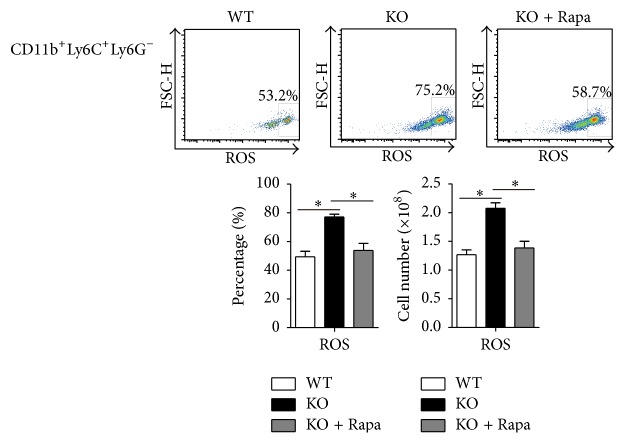
ROS production in monocytes. There was significantly higher ROS production in monocytes from TSC1 KO mice (CD11b^+^Ly6c^+^Ly6G^−^) than from WT mice. The difference was attenuated in monocytes with the Rapa treatment (1.5 mg/kg, four days before treatment) (four mice per group, three representative experiments). ^*∗*^ *P* < 0.05 compared to WT mice or between the indicated groups. *P* values were determined using Student's *t*-tests.

There are previous studies showing that reactive oxygen species (ROS) are involved in the imbalance between Tregs and Th17 cells [19, 20]; therefore, we evaluated the production of ROS by monocytes. In CD11b^+^Ly6c^+^Ly6G^−^ monocytes obtained from TSC1 KO mice, ROS production is higher than in WT mice. This difference was eliminated when monocytes (CD11b^+^Ly6C^+^Ly6G^−^) obtained from TSC1 KO mice were treated by Rapa (Figure 5). Thus, the productivity of ROS is related to activation of mTORC1 in monocytes.

### 3.6. During an* E. coli* Infection, the Antigen Presentation Function of Monocytes Is Weakened. Moreover, the Activation of T Cells and Inflammatory Cytokines Are Declined in TSC1 KO Mice

**Figure 6:**
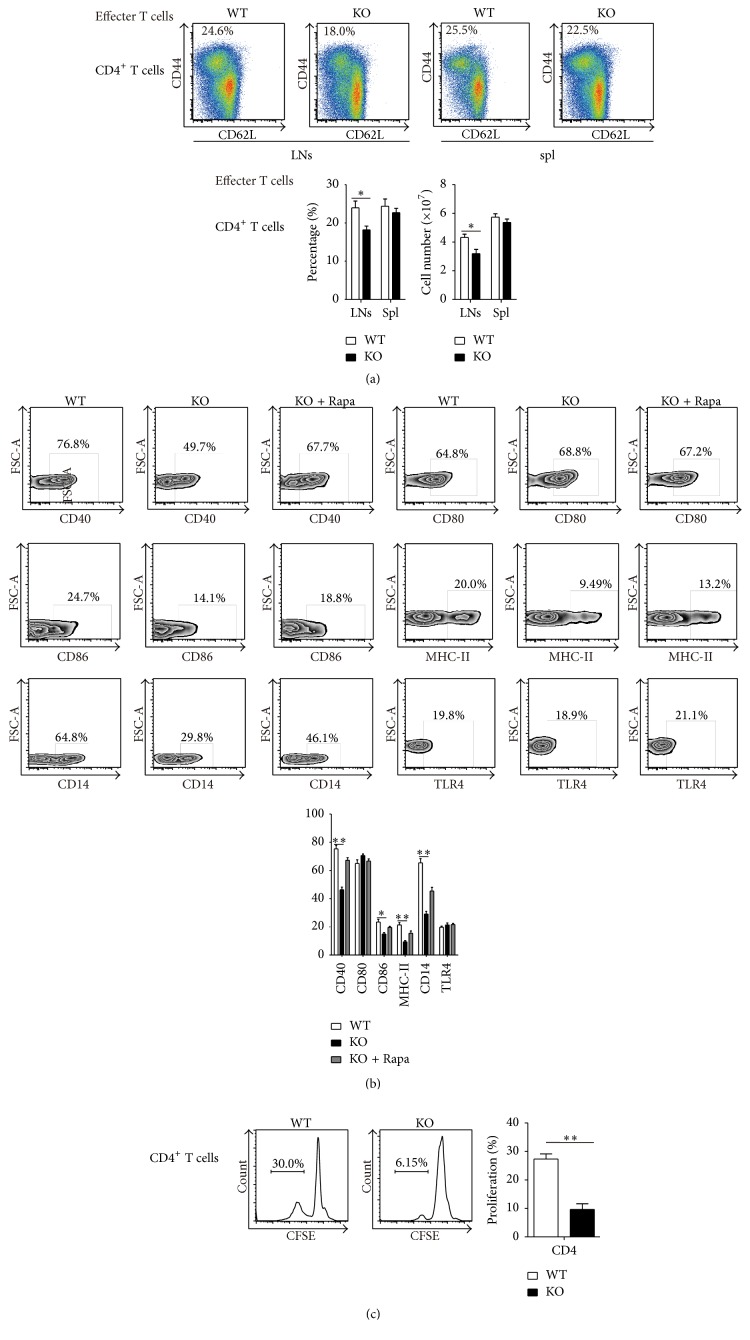
After* E. coli* infection, the expression of activators in monocytes decreased and the activation of effector T cells declined in TSC1 KO mice. (a) 5*∗*10^7^ *E. coli* (ATCC, USA) were injected into each mouse through the tail vein for infection. Eight hours postinfection, the percentage and number of activated effector CD4+ T cells decreased in LNs and spl of TSC1 KO mice compared to WT mice. (b) 5*∗*10^7^ *E. coli* (ATCC, USA) were injected into each mouse through the tail vein for infection. Eight hours postinfection, the expression levels of activators, including CD40, CD80, CD86, MHC-II, and CD14 in monocytes, decrease in TSC1 KO mice than WT mice. (c) 2 × 10^5^ naïve CD4^+^ T cells (CD4^+^CD44^−^CD62^+^) were cocultured with 1 × 10^5^ sorted monocytes (CD11b^+^Ly6G^−^Ly6C^+^) from WT or TSC1 KO mice in DMEM with anti-mouse CD3e (10 *μ*g/mL, BD Biosciences, USA) and CD28 antibody (10 *μ*g/mL, BD Biosciences, USA) in 96-well flat-bottomed plates for 6 days. The proliferation of naïve CD4^+^ T cells cocultured with TSC1 KO monocytes was lower than cells cocultured with the WT group. ^*∗*^ *P* < 0.05 and ^*∗∗*^ *P* < 0.01 compared to WT mice or between the indicated groups. *P* values were determined using Student's *t*-tests.

During bacterial infection, we found that the immune response is impaired in TSC1 KO mice, as evidenced by the greater numbers of bacteria in the blood, lower concentration of proinflammatory cytokines, and greater mortality in TSC1 KO mice. Therefore, we evaluated the percentage and number of CD4^+^ effector T cells in LNs and spleen of WT and TSC1 KO mice. We found that the percentage and number of activated CD4^+^ effector T cells are decreased in TSC1 KO mice when compared to WT mice (Figure 6(a)). Fewer effector T cells in LNs and spleens of TSC1 KO mice could be explained by impaired antigen presentation function of monocytes in TSC1 KO mice. After examination of coactivators in monocytes after bacterial infection, we found reduced expression of coactivators (i.e., CD40, CD86, MHC-II, and CD14) from TSC1 KO mice and WT mice. However, there was no significant difference in CD80 and TLR4 expression (Figure 6(b)). In order to prove if the lower expression of coactivators in TSC1 KO mice caused the less activated CD4^+^ effector T cells, we further determined the proliferation of naïve CD4^+^ T cells cocultured with monocytes acquired from WT or TSC1 KO mice in an in vitro system. The proliferation of naïve CD4^+^ T cells decreased in TSC1 KO mice when compared to WT mice (Figure 6(c)).

## 4. Discussion

In this study, we used a novel model of myeloid-specific* Tsc1* deletion and constitutive mTORC1 activity to elucidate mTORC1 function in monocytes, which partly simulated monocytes behavior during* E. coli* infection. During bacterial infection, we found a greater number of bacteria in the blood, lower concentration of proinflammatory cytokines, and a higher mortality rate in TSC1 KO mice when compared to WT mice. These data suggested that the immune response is impaired in TSC1 KO mice. To explore the relationship between mTORC1 activation in monocytes and an inhibited immune response, we further determined the percentage and number of Tregs, which inhibit immune responses, and found that the numbers of Tregs were significantly higher in TSC1 KO mice compared to WT mice. Meanwhile, the numbers of Th1 were significantly lower in TSC1 KO mice compared to WT mice. Extending the analysis of mTORC1 function in monocytes, we showed that monocytes from TSC1 KO mice had impaired Th17 differentiation, as seen by the reduced percentage and number of Th17 cells in vivo and in vitro. TSC1 KO monocytes produced more IL-6, IL-10, TGF-*β*, and ROS and less IFN-*γ*, IL-1*β*, and TNF-*α* compared to WT mice. Considering the change in cytokine expression by TSC1 KO monocytes, we treated WT monocytes cocultured with naïve CD4^+^ T cells with IL-10 and found that the percentage of Th17 cells decreased. For the balance between Th17 and Tregs [18], this may induce more Tregs in LNs and spleen of TSC1 KO mice and cells coculture experiments. Because Treg numbers increased and CD4^+^ effector T cells decreased, the immune response to bacterial infection seemed to be weakened in TSC1 KO mice. Interestingly, we found lower expression of CD40, CD86, MHC-II, and CD14 in monocytes obtained from TSC1 KO mice and WT mice, suggesting that the antigen presentation function of TSC1 KO monocytes was impaired. Therefore, we cocultured monocytes obtained from TSC1 KO and WT mice with CD4^+^ naïve T cells and found that proliferation of naïve CD4^+^ T cells decreased in TSC1 KO mice when compared to WT mice.

The monocyte is a type of immune cell with numerous functions, such as fighting infection, presenting antigens, and inhibiting immunity. When infected with bacteria, monocytes could phagocytose pathogenic microorganisms or toxins and then present antigen to T cells for priming immune responses [21]. In our study, we found a greater number of bacteria in the blood and higher mortality in TSC1 KO mice compared to WT mice, indicating an impaired ability of defence after activation of mTORC1 in monocytes. In the absence of infection, the percentage and number of Tregs in TSC1 KO mice was higher than that in WT mice, suggesting that constitutive mTORC1 activity may induce more Tregs. During bacterial infection, the percentage and number of activated effector CD4^+^ T cells decreased in TSC1 KO mice. These results indicate that the mTORC1-mediated impaired ability to defend against bacteria might be partially regulated by immune suppression and initiation of immune processes.

In monocytes from TSC1 KO mice, we found increased levels of TGF-*β*, IL-10, and IL-6. IL-6 could enhance the production of IL-1ra and IL-10, leading to inhibition of inflammatory reactions [22, 23]. Previous studies have demonstrated that overexpression of IL-10 and TGF-*β* can induce Tregs [24–26]. However, Tregs cocultured with TSC1 KO monocytes do not proliferate significantly. To explain the decrease of Th17 cells in vivo and in vitro, we hypothesize that the balance of Th17 cells to Tregs may be mediated by mTORC1 activation through the above cytokines. Previous studies showed that IL-6 can promote Th17 differentiation [27]. However, IL-10 can promote Treg proliferation and inhibit Th17 differentiation [28], which is consistent with our data. These results indicate that mTORC1 activation in monocytes can regulate the balance of Tregs to Th17 cells by IL-10 but not IL-6. In addition, antigen stimulation-dependent Th17 differentiation is mediated by increased expression of CD40L on T cells, resulting in increased DC activation and IL-6 production [29, 30]. In our study, the decreased expression of CD40 on monocytes and the possible accompanied decreased expression of CD40L on T cells could together contribute to Th17 differentiation and decline in Tregs induction. This may be another mechanism by which mTORC1 in monocytes controls the balance of Tregs to Th17 cells.

Under hypoxic culture conditions, Tregs generation was shown to be inhibited by Rapa [31]. Macrophages can modulate T cell responses by producing both ROS and Tregs in a ROS-dependent manner [21]. Such changes in ROS production were not observed in TSC1 KO mice treated with Rapa, suggesting that the production of Tregs is ROS-dependent. Interestingly, ROS production by TSC1 KO monocytes increased, but bacterial killing in the KO mice decreased. This may result from the significantly decreased number of effector CD4^+^ T cells and Th1 in TSC1 KO, as the bactericidal effects of CD4^+^ T cells and Th1 are more powerful than ROS. This will require further clarification in the future.

During bacteremia, when monocytes function as APCs, monocytes can induce the expression of several coactivators to enhance their antigen presentation function. For example, CD40 and MHC-II, which are required for antigen presentation to naïve T cells, induce B and T cell activation [32]. With overexpression of CD40 in APCs, CD80 and CD86 can induce and activate T cells, leading to enhanced immune responses [33, 34]. In our study, coactivators (i.e., CD40, CD86, and MHC-II) were decreased in monocytes from TSC1 KO mice when compared to WT mice, suggesting that APC function in monocytes is impaired and results in a lowered ability to induce proliferation of naïve CD4^+^ T cells. Although the role of monocytes during antigen presenting cells is very limited when compared to dendritic cells, it can partly explain the decrease in effector CD4^+^ T cells in TSC1 KO mice and the lower proliferative ability of naïve CD4^+^ T cells cocultured with TSC1 KO monocytes. It is known that Toll-like receptor 4 (TLR4) is activated by lipopolysaccharide (LPS), a component of the outer membrane of Gram-negative bacteria, such as* E. coli,* whose engagement with TLR4 could lead to production of proinflammatory mediators [35]. Binding of LPS to CD14 on plasma membrane initiates TLR4 signaling [36] and transfer of LPS to the TLR4/MD-2 complex triggers the production of proinflammatory cytokines [37]. In our study, we observed decreased expression in CD14, unchanged expression of TLR4, and decreased levels of inflammatory cytokine from monocytes in TSC1 KO mice, indicating that cytokine production is CD14-dependent.

Among several coactivators, CD40 is a key activator expressed on surface of different immune cells, such as endothelial cells, monocytes, B cells, DCs, and keratinocytes [33, 38]. In response to inflammation, the expression of CD40 increased by binding to CD40L, a molecule not expressed under basal conditions but upregulated particularly on T cells and platelets following stimulation [39]. The CD40L-CD40 interaction leads to increased expression of adhesion molecules and release of inflammatory mediators from endothelial cells and leukocytes [40]. In monocytes from TSC1 KO mice, the expression of CD40 is lower than in WT mice after* E. coli* infection, suggesting that the decreased expression of CD40 may lead, at least partially, to an ineffective activation of T cells. The expression of CD40 is regulated by several cytokines, such as TNF-*α*, IFN-*γ*, and IL-1 [41, 42]. The lower expression of TNF-*α*, IFN-*γ*, IL-1, and CD40 in monocytes of TSC1 KO mice implicated that the expression of CD40 may be regulated by activation of mTORC1 through decreased expressions of TNF-*α*, IFN-*γ*, and IL-1.

Interestingly, the changes to inflammatory cytokines and costimulatory molecules in monocytes of TSC1 KO mice do not agree with the results of previous studies [43–45]. The conclusions from these studies conflicted with our results, as macrophages were derived from monocytes. The TSC1/2 complex was found to be an essential repressor for macrophage activation, M1 polarization, and tissue inflammation [44]. The current knowledge on the roles of the TSC1/2-mTOR relationship during innate inflammation are contradictory [46, 47]. Tissue environment itself is a major controller of macrophage phenotype and can influence the expression of many genes regardless of origin [48]. Macrophage resides in inflammatory lesions, but monocytes circulate in peripheral blood. The different tissue environments maybe one of the reasons to explain the different results. In addition, we used* E. coli* infection instead of TLR stimulation (LPS);* E. coli* have many virulence factors, including LPS, capsule, Type 1 fimbriae, and P fimbriae [49]. The results from LPS stimulation do not completely represent the results of the inflammatory events of* E. coli* infection [50]; therefore, the changes to TSC1KO macrophages may be inconsistent with monocytes.

In conclusion, mTORC1 activated monocytes lead to an indirect increase of Tregs and a weakened immune response, which plays a harmful role in bacterial infections. The inhibitor of mTORC1 signal, Rapa, could rescue the harmful effects, which provides a new direction for clinical therapies.

## Supplementary Material

LNs and spleens cells were incubated with antibodies (anti-CD4-APC, anti-CD8-PE/Cy7, anti-CD62L-FITC, and anti-CD44-PerCP/Cy5.5) in the dark for 30min and then washed with staining buffer. Blood samples were treated with red blood cells lysis buffer and washed once with PBS containing 2% FBS. Approximately one million white blood cells were incubated with antibodies (anti-CD11b-PE/Cy7, anti-Ly6G-APC/Cy7, anti-Ly6C-PerCP/Cy5.5, anti-CD80-PE/Cy7, CD40-PE/Cy7, anti-CD86-PE, anti-CD14-FITC, anti-MHC-II-APC, and anti-TLR4-PE) in the dark for 30min and then washed with staining buffer. Cells were measured using a flow cytometer (BD FACSCanto II, USA). The data were analyzed using FlowJo software (Treestar, USA).
