# Supplementary material for: mTORC1-Activated Monocytes Increase Tregs and Inhibit the Immune Response to Bacterial Infections
Source: Mediators Inflamm. 2016 Sep 26;2016:7369351. doi: 10.1155/2016/7369351 (PMC5056273; doi:10.1155/2016/7369351)
Supplement: Supplementary file 1 — LNs and spleens cells were incubated with antibodies (anti-CD4-APC, anti-CD8-PE/Cy7, anti-CD62L-FITC, and anti-CD44-PerCP/Cy5.5) in the dark for 30min and then washed with staining buffer. Blood samples were treated with red blood cells lysis buffer and washed once with PBS containing 2% FBS. Approximately one million white blood cells were incubated with antibodies (anti-CD11b-PE/Cy7, anti-Ly6G-APC/Cy7, anti-Ly6C-PerCP/Cy5.5, anti-CD80-PE/Cy7, CD40-PE/Cy7, anti-CD86-PE, anti-CD14-FITC, anti-MHC-II-APC, and anti-TLR4-PE) in the dark for 30min and then washed with staining buffer. Cells were measured using a flow cytometer (BD FACSCanto II, USA). The data were analyzed using FlowJo software (Treestar, USA). [file 7369351.f1.pdf]

The baseline expression of the markers (CD4, CD8, CD40, CD44, CD86, CD62L, Tregs etc) in WT vs TSC1 KO naïve mice.

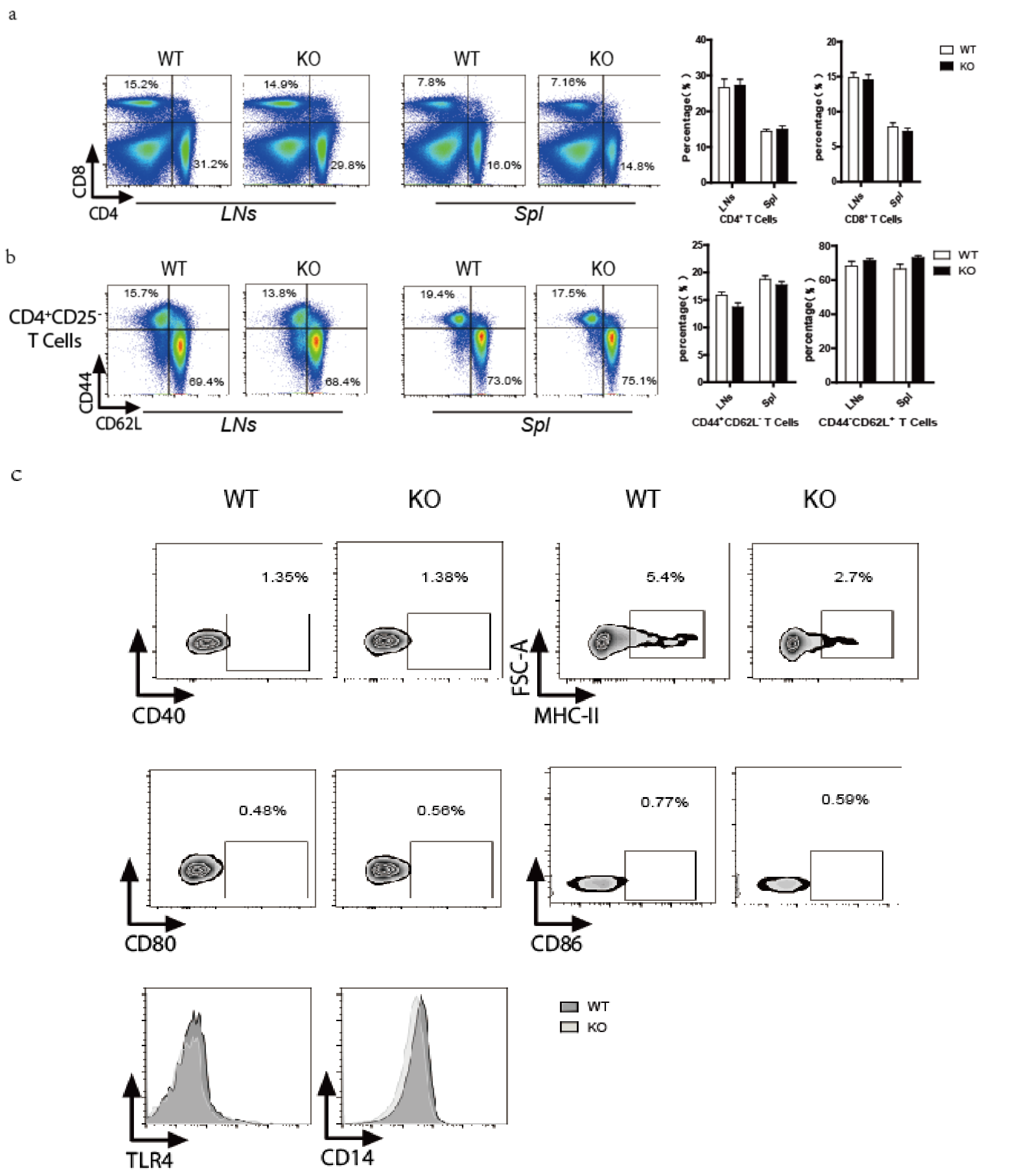

a. The percentage and number of $CD4^+$ or $CD8^+$ T cells in lymph nodes and spleens in WT vs TSC1 KO mice (four mice per group, two representative experiments).

b. The percentage and number of $CD44^+$ $CD62L^-$ or $CD44^-$ $CD62L^+$ T cells in $CD4^+CD25^-$ T cells of lymph nodes and spleens in WT vs TSC1 KO mice (four mice per group, two representative experiments).

c. The percentage of CD40, CD80, CD86, CD14, TLR4 and MHC-II of monocytes in WT vs TSC1 KO mice without bacterial infection (four mice per group, two representative experiments).
